# Alterations to wrist tendon forces following flexor carpi radialis or ulnaris sacrifice: a cadaveric simulator study

**DOI:** 10.1177/1753193418783176

**Published:** 2018-06-27

**Authors:** Darshan S. Shah, Claire Middleton, Sabahat Gurdezi, Maxim D. Horwitz, Angela E. Kedgley

**Affiliations:** 1 Department of Bioengineering, Imperial College London, London, UK; 2 Department of Hand Surgery, Chelsea and Westminster Hospital, London, UK

Dear Sir,

Loss of function of flexor carpi radialis (FCR) or flexor carpi ulnaris (FCU) may result from laceration of the tendon, high median or ulnar nerve injury (Isaacs and Ugwu-Oju, 2016), and, in the case of FCR, use of the tendon for stabilization of the trapeziometacarpal or intercarpal joints. The effects of loss of these flexors have not been evaluated biomechanically. The aim of this study was to understand the importance of the wrist flexors using a physiological wrist simulator, and to observe alterations in wrist tendon forces in the absence of the FCR and FCU.

Nine fresh-frozen cadaveric specimens were used. All soft tissue was resected 5 cm proximal to the wrist, except six muscles, FCR, FCU, extensor carpi radialis longus, extensor carpi radialis brevis (ECRB), extensor carpi ulnaris and abductor pollicis longus (APL), which were dissected at their musculotendinous junctions. Specimens were mounted on a validated physiological wrist simulator (Shah et al., 2017). Motion at the wrist was recreated using linear actuators by applying tensile loads via steel cables sutured to the tendons of the six muscles. Load cells monitored tendon forces, while an eight-camera optical motion capture system was used to obtain the joint angles, through clusters of retroreflective markers fixed to the third metacarpal and the radius. A control strategy, which drove joint kinematics while simultaneously ensuring muscle forces remained within physiological bounds (Shah and Kedgley, 2016), was employed to replicate active wrist motions in real-time. The absence of FCR and FCU were individually simulated in nine and eight specimens, respectively, by switching off the corresponding actuator during the entire range of motion. In the absence of FCR, multiple cycles of 50° flexion–30° extension (FE) and radioulnar deviation (RUD) (15° ulnar to 15° radial) were simulated. In the absence of FCU, complex motions were also simulated, including dart-throwing motion (20° extension with 15° radial deviation to 20° flexion with 15° ulnar deviation), and clockwise (CCD_cw_) and anticlockwise (CCD_acw_) circumduction (combining 30° in FE with 10° RUD) (Shah et al., 2017). Muscle forces were evaluated as a function of joint kinematics, at every 10° in FE and 5° in RUD. Wilcoxon-signed rank tests were performed to identify significant differences in muscle forces with and without FCR and FCU (*p* < 0.05).

**Figure 1.**
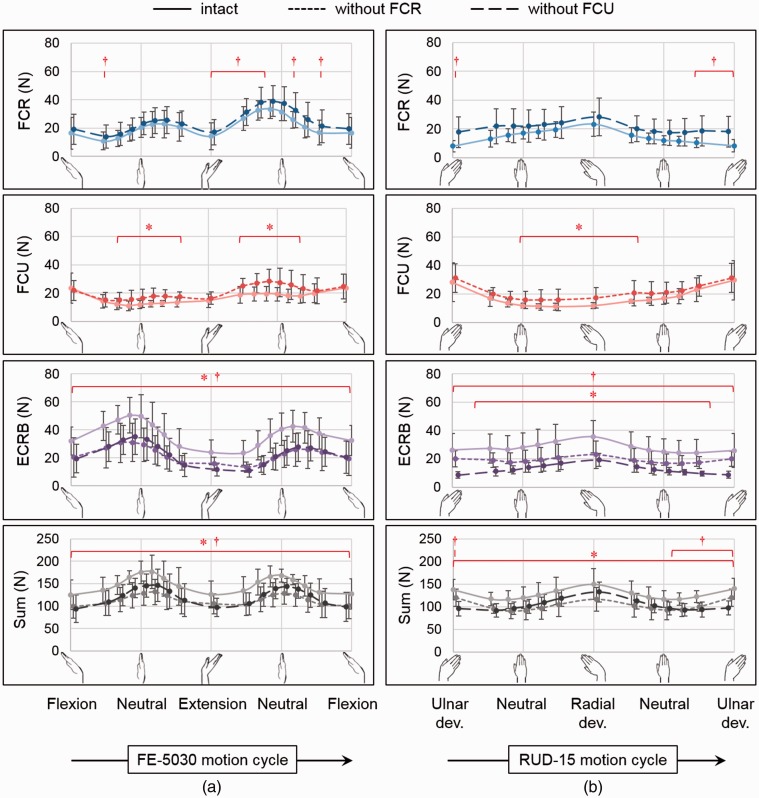
Muscle forces (mean ± standard deviation) for FCR, FCU, ECRB and the sum of all forces, in (a) flexion–extension (FE-5030) and (b) radioulnar deviation (RUD-15) for the intact case (solid line), in the absence of FCR (dotted) and FCU (dashed). The asterisk (*) indicates statistically significant differences between the intact and the FCR-absent groups, while the dagger (†) indicates statistically significant differences between the intact and the FCU-absent groups (*p* < 0.05).

In the absence of FCR, the forces of ECRB and extensor carpi ulnaris were significantly lower throughout FE and RUD, while the APL force was higher throughout FE and radial deviation (Figure 1). The sum of all muscle forces was lower in the absence of FCR throughout FE and RUD, with the decrement being greater than the FCR force in intact specimens. In the absence of the FCU, the ECRB force was significantly lower throughout all motions, while the FCR force was higher from maximum extension to neutral in FE, for ulnar deviation greater than 10° in RUD and dart-throwing motion, and throughout CCD_cw_ and CCD_acw_, except between maximum radial deviation and maximum flexion.

Higher synergist forces observed in the absence of FCR or FCU, especially at joint angles where the force of the intact flexor was high, indicated that the remaining flexors tried to compensate for the loss of FCR or FCU. However, lower antagonist forces for a majority of the range of motion indicated a decrease in co-contraction of the wrist muscles in the absence of FCR or FCU. This finding was reinforced by the lower sum of all muscle forces throughout the ranges of motion in the absence of FCR. The resultant reduction in wrist joint reaction force could contribute to joint weakness. Moreover, while higher forces in synergists could result in muscle strain, fatigue, pain and reduced range of motion, a large reduction in individual muscle forces, like ECRB, could lead to disuse atrophy of the muscle in vivo, as has also been reported in tendon transfers (Castle and Reyman, 1984). These alterations in tendon forces could be important after harvest of FCR for reconstructions, or for tendon transfers employing already weakened donor muscles.

The replication of wrist motions in vitro using only six tendons was a limitation of this study. These tendons were selected since they insert at the base of the metacarpals, thereby primarily affecting the wrist. All muscles contributing to wrist torque, including the extrinsic muscles of the fingers and the thumb, will be considered for future experiments.

In conclusion, the absence of a muscle resulted in a rise in the synergistic forces, with a simultaneous decrease in the antagonistic forces, which was greater than the synergistic force increase. Thus, during any reconstructive procedure that utilizes a tendon, there is likely to be an alteration in the forces exerted by other wrist muscles, the implications of which should be considered during rehabilitation, and in the assessment of ultimate range of motion and functional ability.
